# MiR-130b plays an oncogenic role by repressing PTEN expression in esophageal squamous cell carcinoma cells

**DOI:** 10.1186/s12885-015-1031-5

**Published:** 2015-01-31

**Authors:** Tingting Yu, Risheng Cao, Shuo Li, Mingen Fu, Lihua Ren, Weixu Chen, Hong Zhu, Qiang Zhan, Ruihua Shi

**Affiliations:** 1 Department of Gastroenterology, First Affiliated Hospital of Nanjing Medical University, 300 Guangzhou Road, Nanjing, 210029 China; 2 Department of Gastroenterology, Wuxi People’s Hospital Affiliated with Nanjing Medical University, 299 Qingyang Road, Wuxi, 214023 China; 3 Department of Gastroenterology, Zhongda Hospital Affiliated with Southeast University, 87 Dingjiaqiao Road, Nanjing, 210009 China

**Keywords:** Esophageal squamous cell carcinoma, miR-130b, Oncogenic, PTEN

## Abstract

**Background:**

Esophageal carcinoma is one of the most common malignancies with high cancer-related morbidity and mortality worldwide. MicroRNAs (miRNAs) are a class of small non-coding RNAs that regulate a wide variety of cellular processes, and also play an important role in the development and progression of cancers. In a previous microarray study, we demonstrated that miR-130b was upregulated in esophageal squamous cell carcinoma (ESCC) tissues. However, the biologic functions and the molecular mechanism of miR-130b in ESCC remain to be elucidated.

**Methods:**

qRT-PCR assays were used to quantify miR-130b expression levels in ESCC samples. Novel targets of miR-130b were identified *via* a bioinformatics search and confirmed using a dual-luciferase reporter system. Western blotting and qRT-PCR assays were used to quantify the expression of the target gene *PTEN* (phosphatase and tensin homolog) and the downstream effector, Akt. ESCC cells over- or underexpressing miR-130b were analyzed for *in vitro* biologic functions.

**Results:**

High levels of miR-130b were identified in 20 ESCC samples following comparison with adjacent non-neoplastic tissues. We confirmed that miR-130b interacted with the 3′-untranslated region of *PTEN*, and that an increase in the expression level of miR-130b negatively affected the protein level of PTEN. However, the dysregulation of miR-130b had no obvious impact on *PTEN* mRNA. As Akt is a downstream effector of PTEN, we explored if miR-130b affected Akt expression, and found that miR-130b indirectly regulated the level of phosphorylated Akt, while total Akt protein remained unchanged. Overexpression of miR-130b increased the proliferation of ESCC cells and enhanced their ability to migrate and invade. In contrast, the proliferation, migration, and invasion of ESCC cells were weakened when miR-130b expression was suppressed, which was reversed by *PTEN*-targeted siRNA.

**Conclusion:**

The results indicate that miR-130b plays an oncogenic role in ESCC cells by repressing PTEN expression and Akt phosphorylation, which would be helpful in developing miRNA-based treatments for ESCC.

**Electronic supplementary material:**

The online version of this article (doi:10.1186/s12885-015-1031-5) contains supplementary material, which is available to authorized users.

## Background

Esophageal carcinoma is one of the most common malignancies with high cancer-related morbidity and mortality worldwide. Esophageal squamous cell carcinoma (ESCC) and esophageal adenocarcinoma are the two major histologic types of esophageal carcinoma [1]. The incidence of esophageal carcinoma shows significant regional differences [2]. Notably, the incidence of ESCC is particularly high in the so-called Asian belt, which includes Turkey, northeastern Iran, Kazakhstan, and northern and central China, with more than 100 cases per 100,000 people reported annually [3]. The carcinogenesis of ESCC is proposed as a multistep process. Previous studies have shown that tobacco and alcohol consumption are associated with a higher risk of ESCC [4]. Other risk factors include low socioeconomic status, nutritional deficiencies, excess intake of hot beverages and genetic predisposition [5,6]. Although diagnostic and treatment methods have been developed in recent years, the five-year overall survival of ESCC remains < 14% [1]. Therefore, more specific and sensitive biomarkers for diagnosis and targeted therapy of ESCC are urgently needed.

MicroRNAs (miRNAs) are a class of small non-coding RNAs that are 17–24 nucleotides in length [7]. It is estimated that more than 50% of the miRNAs are located in cancer-related regions and fragile sites of chromosomes [8]. Increasing evidence shows that dysregulation of miRNAs leads to the development and progression of cancers [9]. The up- or downregulation of miRNAs is correlated with specific tumor types; different human cancers have distinct miRNA expression profiles [10]. Studies have shown that aberrant expression of miRNAs affects cell proliferation, apoptosis, metastasis, and sensitivity to chemotherapy and radiotherapy in multiple cancers [11]. This highlights the potential for using expression profiles of specific miRNAs for cancer diagnosis or therapy.

MiR-130b, located at the 22q11 locus [12], plays an oncogenic role in gastric, liver, and endometrial cancers [13-15], and acts as a tumor suppressor in ovarian cancer and thyroid papillary carcinoma [16,17]. A recent study revealed a correlation between miR-130b and prognoses of postoperative esophageal cancer patients, and suggested that upregulation of miR-130b might be an unfavorable factor for ESCC patients [18]. However, the expression levels and the functions of miR-130b in ESCC have not been reported so far. In the present study, we explored the regulation of miRNAs that may be involved in the development and progression of ESCC. We analyzed miRNA expression profiles in matched primary ESCC and corresponding normal esophageal tissues via miRNA microarray analysis and quantitative real-time PCR (qRT-PCR). A dual-luciferase reporter assay was used to verify that phosphatase and tensin homolog (PTEN) is a downstream target of miR-130b. Functional analyses regarding cell proliferation, migration, and invasion were performed to investigate the effect of miR-130b expression on ESCC cells *in vitro*.

## Methods

### Cell lines and clinical samples

Matched ESCC and corresponding adjacent normal esophageal tissues were obtained from 23 patients who underwent esophagectomy for primary ESCC in the First Affiliated Hospital of Nanjing Medical University (Table 1). None of the patients had received preoperative chemotherapy or radiotherapy. All tissue samples were frozen in liquid nitrogen immediately after resection and stored at −80°C. The histologic diagnosis of these tissue samples was confirmed by a pathologist. This study was approved by the Committee for Ethical Review of Research at the First Affiliated Hospital of Nanjing Medical University, and informed consent was obtained from all the patients involved. The human ESCC cell lines, Eca109 and TE13, were purchased from the Cell Bank of the Chinese Academy of Sciences (Shanghai, China).

**Table 1:** **Clinical features of 3 ESCC patients for subjection of Agilent microarray analysis and 20 patients for qRT-PCR validation**

Clinical parameters	Screening set (n = 3)	Validation set (n = 20)
**Age (years)**		
≤60	2	8
>60	1	12
**Gender**		
Male	2	14
Female	1	6
**TNM staging**		
II	2	13
III	1	7
**Tumor diameter**		
≤5 cm	3	16
>5 cm	0	4

### Cell culture and transfection

Eca109 and TE13 cell lines were cultured in Roswell Park Memorial Institute (RPMI)-1640 or Dulbecco’s modified eagle media (Wisent Inc., Quebec, Canada) supplemented with 10% fetal bovine serum (Wisent Inc.) and 1% penicillin-streptomycin (Invitrogen of Thermo Fisher Scientific Inc., Waltham, MA, USA) in a humidified 5% CO_2_ atmosphere at 37°C. The miR-130b mimic (miR-130bm), negative control of miRNA mimic (NC), miR-130b inhibitor (miR-130bi), negative control of miRNA inhibitor (iNC) and *PTEN*-targeted small interfering RNA (siPTEN) were purchased from GenePharma (Shanghai, China) and transfected at a final concentration of 50–100 nM with Lipofectamine 2000 (Invitrogen) according to the manufacturer’s instructions. After 6 h, the cells were returned to normal medium and cultured for an additional 48 (for RNA isolation) or 72 h (for protein extraction). The inhibition efficiency of siPTEN was also determined (Additional file 1).

### MiRNA microarray analysis

Total RNA was isolated from matched ESCC and corresponding normal esophageal epithelium tissues (*n* = 3) with TRIzol (Invitrogen) according to the manufacturer’s instructions. The RNA concentration was measured on a Nanodrop 2000 spectrophotometer (Thermo Fisher Scientific), and the quality of the RNA was examined on a denaturing formaldehyde gel. The RNA was purified (mirVana miRNA Isolation Kit; Ambion of Thermo Fisher Scientific), and 200 ng from each tissue sample was labeled and hybridized (miRNA Complete Labeling and Hyb Kit; Agilent Technologies, Santa Clara, CA, USA) on a miRNA microarray chip (G4870A; Agilent Technologies) encompassing 1205 human and 142 viral miRNA probes. The data were transformed from the hybridization picture with Feature Extraction software v10.7 and integrated using GeneSpring software (Agilent Technologies).

### qRT-PCR

RNA was isolated from ESCC tissue samples and cells as described above and cDNA was synthesized using a TaqMan microRNA Reverse Transcription Kit (Applied Biosystems of Thermo Fisher Scientific) according to the manufacturer’s instructions. The expression level of mature miR-130b in the tissues or the ESCC cells was confirmed by a TaqMan microRNA assay (Applied Biosystems). Levels of *PTEN* mRNA in ESCC cells were quantified using SYBR Green real-time PCR master mix (Applied Biosystems) and specific primers: *PTEN* [Genebank: NM_000314], 5′-TTTGAAGACCATAACCCACCAC-3′ (forward), 5′-ATTACACCAGTTCGTCCCTTTC-3′ (reverse); and glyceraldehyde-3-phosphate dehydrogenase (*GAPDH*) [Genebank: NM_002046], 5′-AGCCTCAAGATCATCAGCAATG-3′ (forward), 5′-TGTGGTCATGAGTCCTTCCACG-3′ (reverse). The relative expression levels of mature miR-130b and *PTEN* mRNA were calculated by the 2^-ΔΔCt^ method and normalized to U6 snRNA and *GAPDH* mRNA levels, respectively. All PCR reactions were performed on a StepOne Plus RT-PCR instrument (Applied Biosystems).

### Dual-luciferase reporter assay

A 59 bp fragment from the 3′-untranslated region (UTR) of *PTEN* containing the putative binding sequences for miR-130b was synthetized cloned into the firefly luciferase pGL3-control vector (Invitrogen). For the reporter assay, Eca109 cells (in logarithmic growth phase) were plated in a 24-well culture plate at a density of 8000 cells/well. The cells in each well were co-transfected with 25 nM of miR-130bm, miR-130bi or NC, 800 ng/μL of miR-130b-pGL3 vector and 0.8 ng/μL of pRL-TK vector (Invitrogen) using Lipofectamine 2000 according to the manufacturer’s instructions. The cell lysates were collected 24 h after transfection. The firefly and Renilla luciferase activities were measured using the dual-luciferase reporter assay system (Promega Corp., Madison, WI, USA). The luciferase activity was detected on a GLOMAX20/20 luminometer (Promega Corp.) and normalized to the Renilla luciferase activity.

### Western blot analysis

Proteins were extracted from harvested Eca109 and TE13 cells using radioimmunoprecipitation assay buffer (Beyotime, Beijing, China) according to the manufacturer’s instructions. Equal amounts of protein lysates (30 μg) were separated by sodium dodecyl sulfate-polyacrylamide gel electrophoresis (SDS-PAGE) on a 10% gel and transferred to a nitrocellulose membrane. The membranes were blocked with 5% non-fat milk in TBST (10 mM Tris–HCl [pH 8.0], 150 mM NaCl, and 0.05% Tween-20) for 1 h at room temperature, and incubated overnight with agitation at 4°C with primary antibodies against human PTEN, Akt, p-Akt (1:1000; Cell Signaling Technology Inc., Danvers, MA, USA), and GAPDH (1:6000; Bioworld, Visalia, CA, USA). The membranes were washed and incubated with goat anti-rabbit IgG secondary antibody (1:5000; Santa Cruz Biotechnology, Dallas, TX, USA) for 1 h. The immunoreactivity was assessed on a gel-imaging analyzer (Bio-Rad Laboratories Inc., Hercules, CA, USA). The band densities of PTEN, Akt and p-Akt were measured using Image Lab software (Bio-Rad Laboratories, Inc.) and normalized to GAPDH.

### Cell proliferation assay

The transfected cells that were in the logarithmic growth phase were seeded into 96-well plates at a cell density of 2000 cells/well for Eca109 and 5000 cells/well for TE13. Five wells were set up for each transfected group. Cell counting kit-8 (CCK-8) solution (10 μL) was added to one well in each group every 24 h for four consecutive days, incubated at 37°C for 2 h, and the optical density was measured at 450 nm on a microplate reader (Thermo Fisher Scientific Inc.). The proliferation curve was determined using the average optical density values of each group.

### Colony formation assay

After transfection, approximately 300 Eca109 cells were added to each well of 6-well plates and incubated at 37°C for 2 wk. The culture medium was removed twice a week and replaced with fresh medium. On day 14, the cells were washed twice with PBS, fixed with methanol for 30 min and stained with crystal violet staining solution (Beyotime, Beijing, China) for 30 min. The dishes were washed with PBS three times and air-dried. The colonies that contained > 50 cells were counted and the colony forming efficiency was calculated.

### Anchorage-independent growth ability assay in soft agar

Two thousand Eca109 cells were trypsinized and suspended in 2 mL complete medium plus 0.3% agar (Becton, Dickinson and Co., Franklin Lakes, NJ, USA). The agar–cell mixture was plated on top of a bottom layer with 0.6% complete medium agar mixture. After 2 wk, viable colonies that contained ≥ 50 cells or were > 50 μm in diameter were counted.

### Transwell migration and invasion assays

For the cell migration assay, Transwell chambers (EMD Millipore, Billerica, MA, USA) with 8.0-μm-pore-size basement membranes were placed into a 24-well culture plate. For the cell invasion assay, the upper surface of the membrane was evenly covered with 50 μL of 1 mg/mL Matrigel (Becton, Dickinson and Co.). Briefly, 200 μL serum-free cell suspension containing 1 × 10^5^ Eca109 cells was added to the upper compartment of the chamber, and the lower compartment was filled with RPMI-1640 medium containing 10% fetal bovine serum as a chemoattractant. After incubation at 37°C for 48 h, the cells were fixed with 75% ethanol and stained with crystal violet staining solution. Non-migrating cells on the upper surface of the membrane were removed with cotton swabs. The migratory and invasive cells in each chamber were counted from at least five randomly chosen high power fields.

### Statistical analysis

Statistical analyses were performed using SPSS 17.0 software (SPSS Inc., Chicago, IL, USA). The expression of miR-130b in ESCC and matched normal esophageal tissues was analyzed by paired-samples *t*-test, and independent-samples *t*-tests were used for other data analyses; *P* < 0.05 was considered statistically significant. All data from this study were obtained from at least three independent experiments and are expressed as mean ± SEM.

## Results

### MiR-130b is overexpressed in ESCC tissues

**Figure 1:**
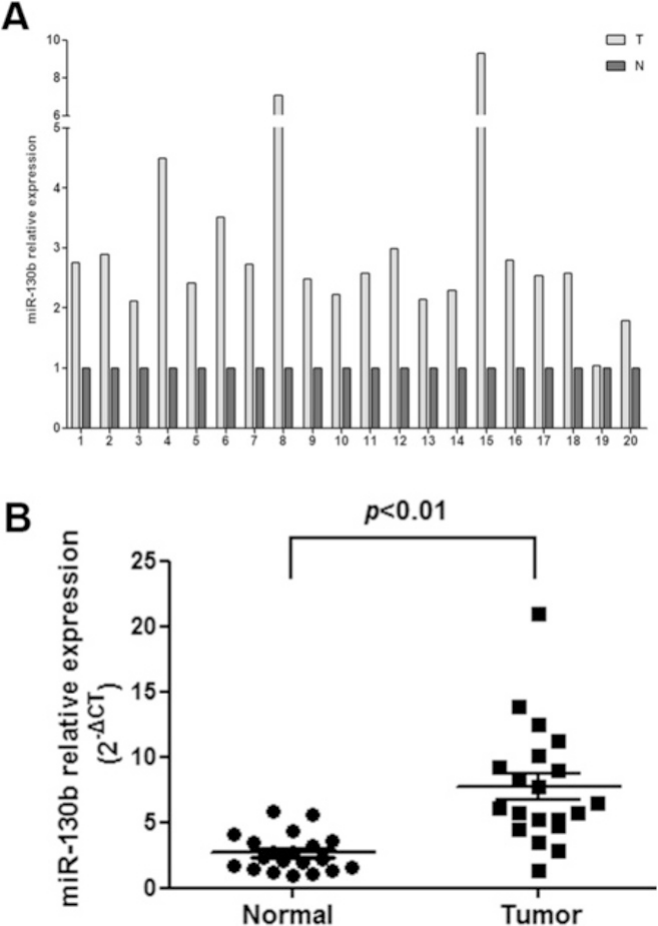
**Expression of mature miR-130b in ESCC tissue specimens.** The expression of miR-130b was detected by TaqMan qRT-PCR in 20 ESCC and matched normal esophageal tissue samples. The expression of miR-130b is presented on a 2^-ΔCT^ (×100) scale and normalized against the endogenous control U6. **(A)** Paired comparison of miR-130b expression in ESCC tissue and matched normal tissue. **(B)** Scatter plots with merged data.

To determine the effect of miRNAs on the development and progression of ESCC, we first performed a miRNA microarray analysis (cutoff, > 2.0 fold-change). The results of the microarray analysis, which had been deposited to Gene Expression Omnibus (GEO) database [GEO: GSE59973], showed that 51 miRNAs were significantly overexpressed in ESCC tissues when compared with normal esophageal tissues, including miR-130b, which showed a fivefold-change over the normal esophageal tissues (Table 2). Quantification of miR-130b expression in 20 ESCC and matched normal esophageal tissue samples confirmed the microarray results (Figure 1).

**Table 2:** **The miRNA microarray data of highly upregulated (fold-change > 5.0) miRNAs in esophageal squamous cell carcinoma (ESCC) tissues compared with normal esophageal tissues**

Up-regulated miRNAs	Fold-change	*P*
miR-7	10.91	0.005
miR-196b	7.67	0.030
miR-424	6.75	0.008
miR-18b	6.67	0.002
miR-223	6.31	0.003
miR-130b	5.18	0.005

### *PTEN* is a target of miR-130b

**Figure 2:**
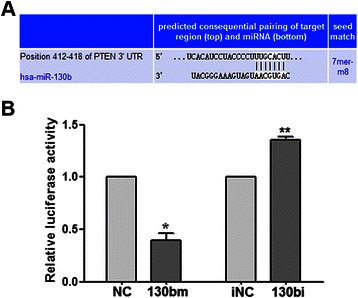
**Identification of PTEN as target of miR-130b. (A)** Bioinformatic prediction by TargetScan indicated that *PTEN* mRNA contains a putative miR-130b binding region, located at 412–418 nt of 3′-UTR. **(B)** A dual-luciferase reporter assay was used to confirm the interaction of miR-130b with *PTEN*. A fragment of miR-130b that was predicted to bind to the *PTEN* 3′-UTR was cloned into firefly luciferase pGL3-control vector. The Renilla luciferase plasmid was co-transfected for normalizing luciferase activity. *P < 0.05, **P < 0.01 *vs.* corresponding controls.

By integrating the search results of TargetScan and miRDB programs (Figure 2A), we identified the tumor suppressor gene *PTEN* as a potential target of miR-130b. A dual-luciferase reporter assay was performed to verify that the 3′-UTR of *PTEN* mRNA is a direct target of miR-130b. The luciferase activity of Eca109 cells transfected with miR-130bm was reduced by approximately 60% when compared to the NC transfected cells, whereas miR-130bi increased the activity (Figure 2B).

### MiR-130b regulates expression of PTEN and phosphorylation of Akt

**Figure 3:**
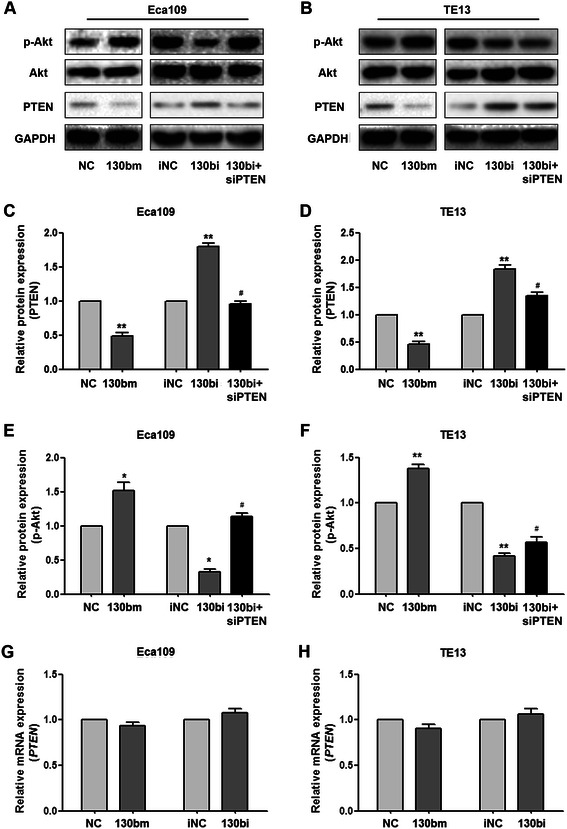
**MiR-130b regulates the expression of PTEN and phosphorylation of Akt.** Eca109 and TE13 cells were transfected with miR-130b mimic (130bm group) or inhibitor (130bi group); siPTEN was also co-transfected with the miR-130b inhibitor to decrease PTEN expression (130bi + siPTEN group). Protein expression of PTEN, p-Akt and total Akt in Eca109 **(A, C, E)** and TE13 cells **(B, D, F)** was determined by Western blot analysis. *PTEN* mRNA expression was detected by SYBR Green real-time PCR at 24 h following transfection of Eca109 **(G)** and TE13 **(H)** cell lines. The values were normalized to the corresponding controls. GAPDH was the endogenous control. *P < 0.05, **P < 0.01 *vs.* corresponding controls; #*P* < 0.05 *vs.* 130bi group.

To investigate the possible underlying molecular mechanism of miR-130b in ESCC tissues, Eca109 and TE13 cells were transfected with miR-130bm and miR-130bi to increase and decrease miR-130b expression, respectively. PTEN targeted siRNA was also co-transfected with miR-130bi to identify the effect of PTEN protein expression. Western blotting (Figure 3A–D) and qRT-PCR (Figure 3G, H) analyses showed that miR-130b negatively regulated levels of PTEN protein but not mRNA, indicating regulation at the translational level.

PTEN is the main negative regulator of the phosphatidylinositol 3 kinase/Akt pathway [19]. Therefore, we explored whether miR-130b regulation of PTEN protein levels affects Akt phosphorylation. Western blotting showed that upregulation of miR-130b was accompanied by an increase in the levels of phosphorylated Akt (p-Akt) (Figure 3A,B,E,F). Conversely, p-Akt levels decreased when the expression of miR-130b was suppressed. Interestingly, total Akt protein levels remained unchanged, indicating that miR-130b regulates the phosphorylation of Akt, rather than the protein expression.

### MiR-130b regulates the proliferation of ESCC cells

**Figure 4:**
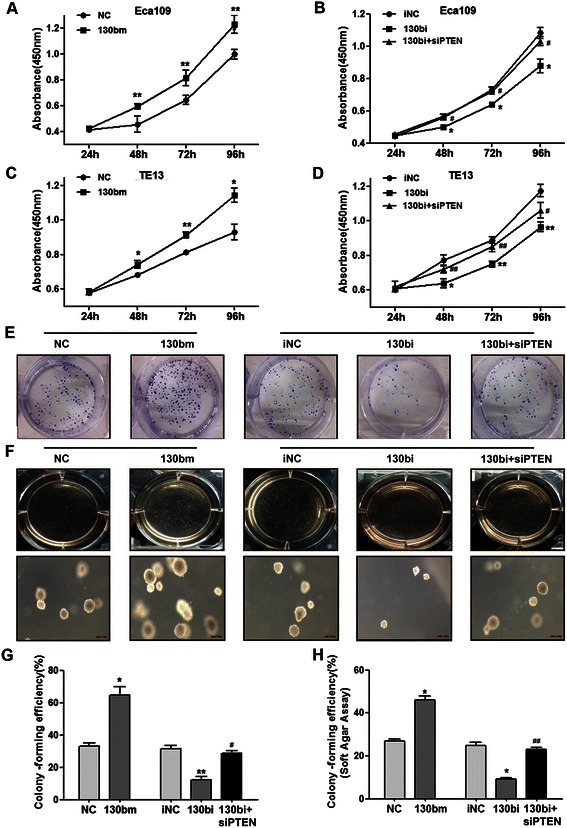
**Effect of miR-130b on the proliferation of ESCC cells*in vitro.*** The viability of Eca109 and TE13 cells transfected with miR-130b mimic, negative control, miR-130b inhibitor, inhibitor negative control or siPTEN was detected by CCK-8 at 24, 48, 72 and 96 h. The absorbance was measured at 450 nm. **(A, B)** Proliferation curves of Eca109 cells. **(C, D)** Proliferation curves of TE13 cells. For colony formation **(E, G)** and soft-agar **(F, H)** assays in Eca109 cells, the following calculation was performed: colony-forming efficiency (%) = the number of colonies (>50 cells or larger than 50 μm)/the number of cells plated per well. The photographs were captured from digital camera or microscope at 40× magnification, in which the size bar was representative of 200 μm. The results are expressed as mean ± SEM of three independent experiments. *P < 0.05, **P < 0.01 *vs.* corresponding controls; #*P* < 0.05, ##*P* < 0.01 *vs.* 130bi group.

We used CCK-8 and colony formation assays to examine the proliferative ability of ESCC cells. Cell growth curves were constructed from CCK-8 absorbances in Eca109 and TE13 cells at 24, 48, 72 and 96 h, showing that the rate of cell growth in the miR-130bm transfected group was significantly increased compared with NC groups (*P* < 0.05) (Figure 4A-D). In contrast, the viability of ESCC cells was inhibited following transfection with miR-130bi, which was reversed by co-transfection with a *PTEN*-targeted small interfering RNA. Similar results were obtained from colony formation and soft-agar assays in Eca109 cells (Figure 4E–H).

### MiR-130b promotes ESCC cell migration and invasion *in vitro*

**Figure 5:**
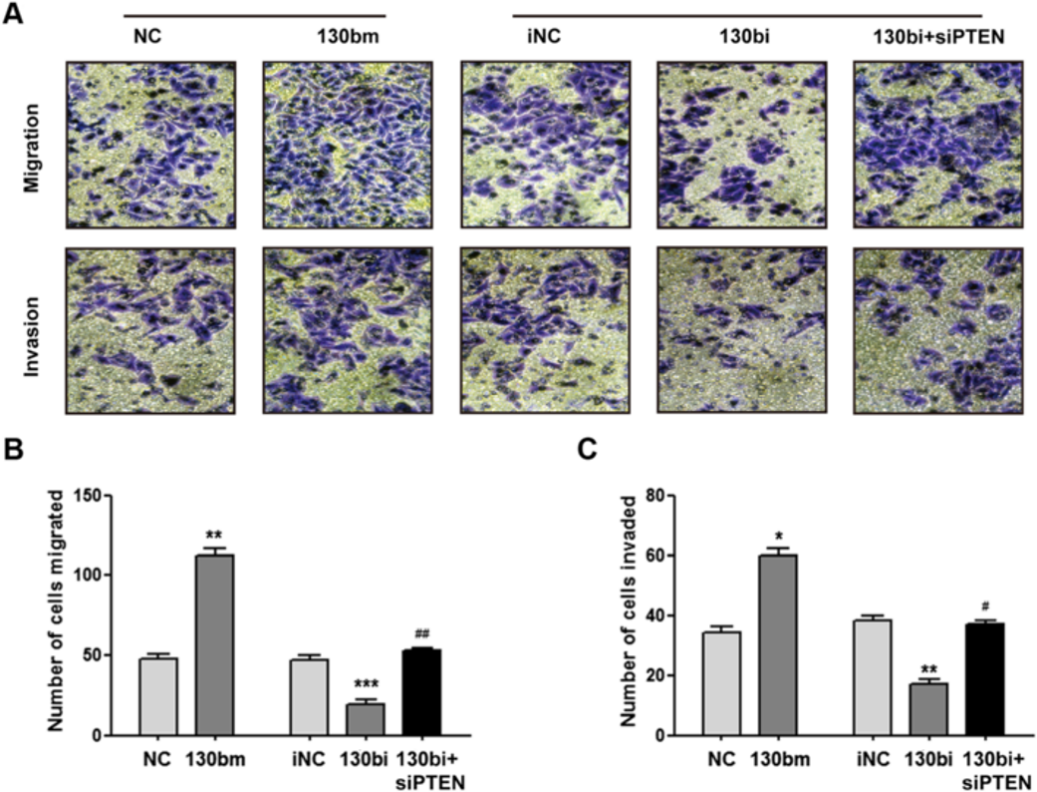
**MiR-130b regulates the migration and invasion of ESCC cells.** 1 × 10^5^ Eca109 cells were added to the Transwell inserts. For the invasion assay, the wells were covered with 50 μL of 1 mg/mL Matrigel. Cells were transfected with miR-130b mimic, negative control, miR-130b inhibitor, inhibitor negative control or siPTEN. The migratory or invasive cells were counted from five random areas at 200× magnification. **(A)** Cell images captured by microscopy. **(B)** Histogram of cell migration results. **(C)** Histogram of cell invasion results. The results are presented as mean ± SEM from three independent experiments. *P < 0.05, **P < 0.01, ***P < 0.001 *vs.* corresponding controls; #*P* < 0.05, ##*P* < 0.01 *vs.* 130bi group.

Tumor cell migration and invasion are one of the leading causes of tumor metastasis. Therefore, we addressed whether miR-130b affects the ability of Eca109 cells to migrate and invade. Overexpression of miR-130b significantly increased the number of cells capable of migration and invasion (*P* < 0.05) (Figure 5). Moreover, inhibition of PTEN expression rescued the decreased cell migration and invasion capacities induced by miR-130bi.

## Discussion

A better understanding of the mechanisms underlying the carcinogenesis of ESCC is important for developing more effective diagnostic and therapeutic strategies. As studies on ESCC advanced, they were no longer limited to oncogenes or tumor suppressor genes, and extended to include entire signaling pathways and gene interaction networks. MiRNAs affect a variety of cellular pathways, and a single miRNA can target multiple genes [20,21]. This specific characteristic of miRNAs has made them a topic of intense research in recent years.

It is estimated that miRNAs regulate the expression of 30–60% of human genes [20,22]. MiRNAs regulate gene expression at the transcriptional or translational level by binding to the 3′-UTR of mRNAs [23]. Whether a miRNA acts as a tumor promoter or repressor depends on the gene that it interacts with [24]. Previous studies have reported associations of miR-130b with some types of solid tumors. For example, miR-130b is significantly overexpressed in gastric cancers, which increases cell viability and decreases the expression of runt related transcription factor 3 gene and Bim during transforming growth factor β-mediated apoptosis [13]. Another study showed that miR-130b promotes CD133^+^ liver tumor-initiated cell growth and self-renewal *in vitro* and *in vivo* via tumor protein 53-induced nuclear protein 1 [15]. The expression of miR-130b is increased in hyperplastic endometrium and increases even further in endometrial cancer, which, along with DICER1 dysfunction, leads to tumor aggression both *in vitro* and *in vivo* [14]. In contrast, miR-130b is downregulated in ovarian cancer and papillary thyroid carcinoma, and its expression is inversely correlated with the tumor aggressiveness and multidrug resistance in these cancers [16,17].

Recently, the first research report showing a correlation between miR-130b and ESCC in a study comparing ESCC patients with the same tumor node metastasis stage but different prognoses [18]. Their results demonstrated that miR-130b was significantly downregulated in the paracancerous normal esophageal mucous membranes of patients with a good prognosis. Therefore, miR-130b may play disparate tumor-associated roles that are likely related to differences in tumor types and genes targeted. To date, no published study has focused on the functions and molecular mechanisms of miR-130b in ESCC. In our study, miRNA microarray analysis and qRT-PCR confirmed that miR-130b is overexpressed in ESCC tissues. Furthermore, increased expression of miR-130b promoted proliferation, migration and invasion *in vitro*, whereas these abilities were weakened when miR-130b was inhibited.

PTEN is located at 10q23.3 and encodes a dual-specificity phosphatase with lipid and protein phosphatase activities. PTEN dephosphorylates PI(3,4,5)P3, an important activator of Akt [25], which controls a variety of cellular processes, such as survival, cell cycle progression, metabolism, and angiogenesis [26]. Inhibition of PTEN results in increased levels of activated p-Akt [19]. Decreased expression of *PTEN* in ESCC is correlated with prognosis [27,28]. The proliferation of ESCC cells *in vitro* and *in vivo* is promoted by transfections with *PTEN* expression vectors [29]. Moreover, PTEN is frequently mutated or there is loss of heterozygosity in multiple malignancies, but these occur rarely in ESCC [30,31]. Several studies have reported that certain miRNAs can directly target PTEN [25]. MiR-21 was among the earliest miRNAs for which it was confirmed that its suppression increased PTEN levels and decreased tumor cell proliferation, migration, and invasion in human hepatocellular cancer [32]. Subsequent studies indicated that PTEN is a potential target of miR-221/222, miR-22 and miR-144 in different malignancies [33-35]. Our study shows that miR-130b binds the 3′-UTR of *PTEN* mRNA to downregulate PTEN protein expression, thus promoting Akt phosphorylation. Moreover, silencing of PTEN expression reversed the effect of miR-130bi on cell proliferation, invasion and migration.

## Conclusion

In summary, our study reveals that miR-130b is overexpressed in ESCC tissues. *PTEN* is a target of miR-130b, which regulates expression at the translational level. Suppression of PTEN results in increased p-Akt, promoting the proliferation, migration and invasion of ESCC cells *in vitro*. Our findings suggest that miR-130b may be a new target for ESCC diagnosis and therapy. However, the clinical sample size in this study was limited and the investigation of miR-130b function in ESCC was restricted to *in vitro* experiments. To further confirm the role of miR-130b in carcinogenesis, the expression level of miR-130b should be compared among a larger number of clinical specimens with different pathologic gradings, and also in animal models.

## Additional file

Additional file 1: **The inhibition efficiency of PTEN targeted siRNA.** Eca109 and TE13 cells were transfected with PTEN targeted siRNA (siPTEN) and negative control (NC) as described in the Methods section. Western blot analysis and qRT-PCR were performed to determine the protein **(A)** and mRNA **(B)** expression of PTEN. GAPDH was the endogenous control. The results are expressed as mean ± SEM of three independent experiments. ****P* < 0.001 *vs.* corresponding controls.

## Abbreviations

CCK-8: Cell counting kit-8
ESCC: Esophageal squamous cell carcinoma
GAPDH: Glyceraldehyde-3-phosphate dehydrogenase
iNC: Negative control of miRNA inhibitor
miR-130bi: miR-130b inhibitor
miR-130bm: miR-130b mimic
miRNA: microRNA
NC: Negative control of miRNA mimic
PTEN: Phosphatase and tensin homolog
p-Akt: Phosphorylated Akt
qRT-PCR: Quantitative real-time PCR
RPMI: Roswell Park Memorial Institute
siPTEN: *PTEN*-targeted small interfering RNA
UTR: Untranslated region
