# Supplementary figures and images for: MiR-130b plays an oncogenic role by repressing PTEN expression in esophageal squamous cell carcinoma cells
Source: BMC Cancer. 2015 Jan 31;15:29. doi: 10.1186/s12885-015-1031-5 (PMC4318221; doi:10.1186/s12885-015-1031-5)

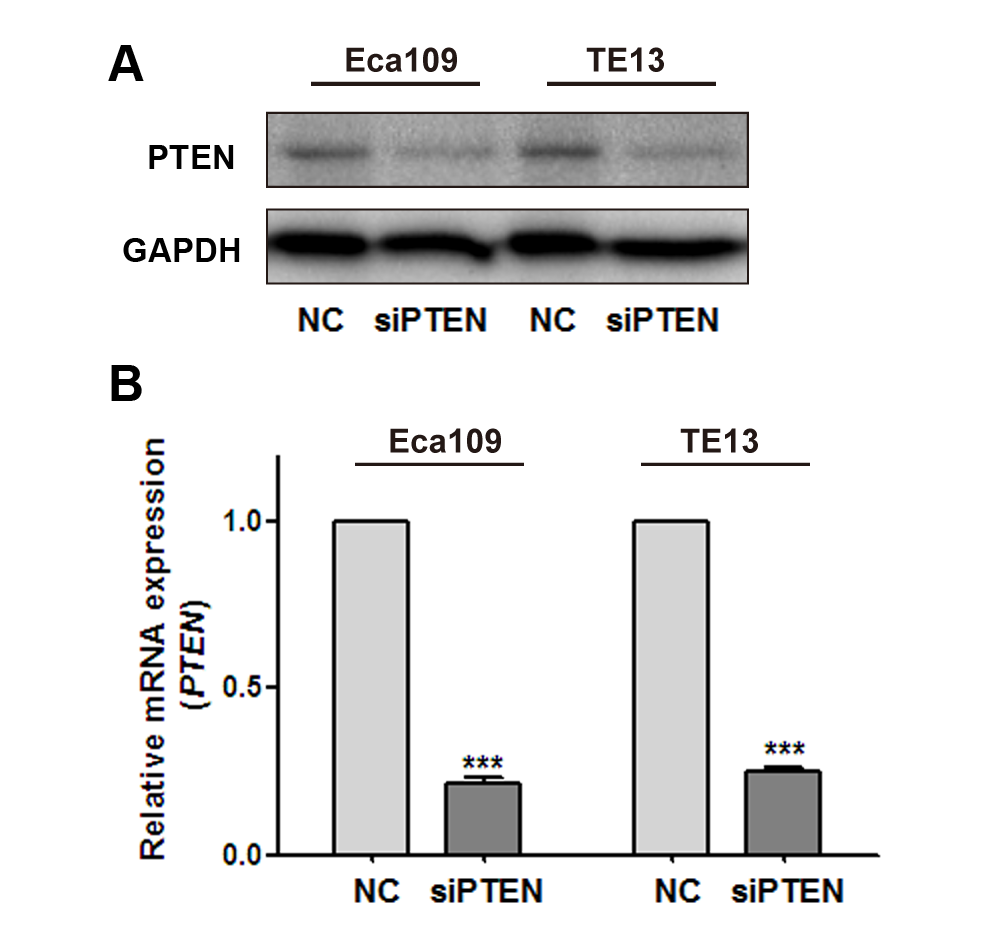

Supplement: Additional file 1: — The inhibition efficiency of PTEN targeted siRNA. Eca109 and TE13 cells were transfected with PTEN targeted siRNA (siPTEN) and negative control (NC) as described in the Methods section. Western blot analysis and qRT-PCR were performed to determine the protein (A) and mRNA (B) expression of PTEN. GAPDH was the endogenous control. The results are expressed as mean ± SEM of three independent experiments. ***P < 0.001 vs. corresponding controls. [file 12885_2015_1031_MOESM1_ESM.tiff]
